# Cardio-metabolic risk among healthcare providers: A latent profile study

**DOI:** 10.34172/jcvtr.025.33541

**Published:** 2025-12-17

**Authors:** Parya Esmaeili, Sayyed M. Haybatollahi, Neda Roshanravan, Samad Ghaffari, Naimeh Mesri Alamdari, Saeed Mousavi, Mohammad Asghari-Jafarabadi

**Affiliations:** ^1^Liver and Gastrointestinal Diseases Research Center, Tabriz University of Medical Sciences, Tabriz, Iran; ^2^Department of Epidemiology and Biostatistics, Faculty of Health, Tabriz University of Medical Sciences, Tabriz, Iran; ^3^Department of People and Performance, Manchester Metropolitan University, Manchester, UK; ^4^Cardiovascular Research Center, Tabriz University of Medical Sciences, Tabriz, Iran; ^5^Endocrine Research Center, Tabriz University of Medical Sciences, Tabriz, Iran; ^6^Road Traffic Injury Research Center, Tabriz University of Medical Sciences, Tabriz, Iran; ^7^Cabrini Research, Cabrini Health, 154 Wattletree Rd, Malvern, VIC 3144, Australia; ^8^School of Public Health and Preventive Medicine, Faculty of Medicine, Nursing and Health Sciences, Monash University, Melbourne, VIC, 3004, Australia; ^9^Department of Psychiatry, School of Clinical Sciences, Faculty of Medicine, Nursing and Health Sciences, Monash University, Clayton, VIC, 3168, Australia

**Keywords:** Cardio-metabolic disease, Risk factors, Latent profile analysis, Healthcare providers, Preventive health

## Abstract

**Introduction:**

Cardio-metabolic disease (CMetD) is a prevalent health issue among healthcare professionals, and suboptimal management of metabolic disorders places a burden on the healthcare system.

**Methods:**

The present study aimed to cluster the participants based on risk factors for the CMetDs using Latent Profile Analysis (LPA). This study was conducted on 500 healthcare providers, aged 18 to 75 years at Tabriz University of Medical Sciences, Tabriz, Iran. LPA was used to explore the latent risk profiles based on age, blood pressure (BP), lipid profile, insulin, body mass index (BMI), and waist circumference.

**Results:**

The individuals were classified into three LPA-driven profiles: low (42.4%), intermediate (21.8%), and high (35.8%). The high-risk profile found in older age and higher BMI, insulin, fasting blood glucose (FBS), as well as higher levels of high-density lipoprotein (HDL) cholesterol, low-density lipoprotein (LDL) cholesterol, total cholesterol, and triglyceride. Furthermore, in the intermediate risk profile, elevated levels of systolic/diastolic BP and waist circumference were associated with higher levels of risk. Haemoglobin and hematocrit levels were significant predictors of low and intermediate latent profiles. Higher levels of hemoglobin and hematocrit were associated with lower odds of being in low and intermediate latent profiles, compared to the high-risk profile (all *P*<0.05).

**Conclusion:**

LPA-derived latent profiles and the specific predictors of profiles help find control and prevention measures in CMetDs; older individuals with poorer lipid profiles, and, elevated insulin, triglyceride, FBS, BP, and BMI levels should be screened more carefully.

## Introduction

 Cardio-metabolic diseases (CMetDs), which are classed as non-communicable diseases (NCDs), are non-transmissible diseases with serious health and economic burden worldwide.^1-4^ CMetDs include a range of diseases such as obesity-related traits, metabolic syndrome, cardiovascular disease (CVD), and type 2 diabetes.^5-7^ According to the WHO, CVD is one of the most critical threats to human health worldwide.^8,9^ Due to the lack of systematic management of diagnostic tests for CVD, metabolic disorders often remain undiagnosed, placing a burden on the healthcare syste.^10,11^ CVD accounted for 17.9 million of all deaths in the world in 2016, and the rate is expected to increase to 23.6 million by 2030.^12,13^ CVD was among the first causes of mortality in Iran in 2023 with a rate of 28.7%.^14^ It is predicted that about 58% of the world’s population will be overweight or obese,^15^ and the number of people with diabetes will increase to 366 million by 2030.^16^

 CMetDs are affected by various risk factors including hypertension, dyslipidaemia, obesity, diabetes, and high triglyceride levels are amongst the known underlying risk factors for CMetDs.^17-20^ The seventy of CMetD risk factors vary among individuals depending on their age, sexual maturity, and family history of the disease.^21,22^ Epidemiological evidence suggests that risk factors show up in childhood and persist into adulthood, and more importantly, the incidence of the disease increases with age.^18,19,22,23^ In addition, carbohydrate diet,^7^ physical activity,^18^ sleep duration,^20^ prolonged sitting,^7^ and reducing smoking and alcohol use^3^ are all important factors associated with the risks of CMetDs. Nevertheless, it is essential to recognize that not all individuals exhibiting one or a combination of the aforementioned risk factors fall within the susceptible population for CMetDs. The existing body of scientific literature underscores the need for further investigation through the implementation of profile-based analysis. This analytical approach involves categorizing the population according to diverse permutations of risk factors associated with CMetDs. By employing this method, a more comprehensive and nuanced perspective can be attained in the identification of high-risk groups and the development of potential health intervention programs.^24^ Clustering participants based on the risk factors of CMetDs helps to identify what groups of people are more vulnerable to CMetDs and what groups are not CMetDs. This study aimed to cluster the participants based on their numeric and categorical risk factors for the CMetDs using Latent Profile Analysis (LPA). In this paper, we use baseline data collected by the AZAR cohort study^25^ to explore the risk factors associated with CMetDs and investigate interventions planned to prevent the disease.

## Materials and Methods

###  Study design, aims, and procedures

 We studied a cohort of individuals that are part of a larger prospective epidemiological research in Iran, the AZAR Cohort Study. The purpose of the AZAR cohort is to evaluate 3000 participants, including healthcare employees in hospitals, schools, and health networks of TBZMED. Further details can be found elsewhere.^26^ The current study used baseline data collated for healthcare providers as part of the AZAR cohort study, which was conducted by the liver and gastrointestinal diseases research centre of Tabriz University of Medical Sciences (TBZMED).^25^ A total of 500 healthcare providers participated in baseline in 2020. Our baseline assessment consisted of a face-to-face health interview. Health examinations were also used on some established risk factors.

###  Eligibility criteria

 Participants of this study were full-time and long-term employees, aged 18 to 75 years, non-pregnant or non-breast feeders, and those who were not planning to retire within the next five years. Healthcare providers who reported a history of debilitating psychiatric disorders or physical illnesses by a health professional were excluded from the study. Indeed, all eligible healthcare providers, of TBZMED participated in this study.

###  Ethical considerations

 All participants provided written informed consent, and the institutional review board (IRB) of TBZMED approved the protocol of this study (IR.TBZMED.REC.1400.1006).

###  Main variables

 Systolic/diastolic blood pressure (BP), fasting blood glucose (FBS), insulin, high-density lipoprotein (HDL) cholesterol,low-density lipoprotein (LDL) cholesterol, total cholesterol, triglycerides, body mass index (BMI), and waist circumference were assessed and used in our LPA model.

###  Demographic characteristics of the participants

 We used a checklist to collect the demographic information, including age (years), sex (1: male, 2: female), marital status (1: single, 2: married, 3: widow, 4: divorce, 4: other), and educational level (1: illiterate, 2: primary, 3: secondary, 4: high school graduated, 5: associate degree, 6: bachelor, 7: master, 8: PhD). Moreover, lifestyle information including history of smoking (1: past or current, 2: never), history of alcohol (1: unknown, 2: daily, 3: occasionally), history of diabetes (1: yes, 2: no), and history of hypertension (1: yes, 2: no) also were collected by the checklist.

###  Measurements

 Body mass index was calculated using the standard formula, i.e., weight (kg)/height2 (m).^25^

 Waist circumference was measured according to the USA National Institutes of Health (NIH) guidelines.^27^

 BPwas measured by a trained nurse twice at a two-minute interval. Each arm was measured in a sitting position using a mercury sphygmomanometer after 10 minutes of rest (Rudolf Richter, DE-72417, Germany).

 Lipid profiles (total cholesterol, high-density lipoprotein cholesterol (HDL-cholesterol), and triglycerides) were assessed using serum samples, which were analyzed using Miura one automated equipment (I.S.E., Rome, Italy) and a commercial DiaSys kit (DiaSys Diagnostic Systems, Hamburg, Germany).^28^

 Low-density lipoprotein cholesterol (LDL-cholesterol) was calculated according to the Fried Ewald equation.^29^ Metabolic syndrome is a cluster of risk factors that include central obesity, high blood pressure, high blood sugar, and abnormal cholesterol levels. It is a significant predictor of cardiovascular disease, diabetes, and other chronic health conditions. The diagnosis of metabolic syndrome is based on the presence of three or more of these risk factors.^30^

 Insulin was measured using commercial radio-immune assay (RIA) kits (1989: charcoal-coated RIA; 2004: LINCO Research Inc, Missouri, USA).^31^

 Hematocrit was measured using (EKF Diagnostics UltraCrit ^TM^ Hematocr, USA). A photometric device (HemoCue® Hb 201 + ) was used to determine the hemoglobin content of the blood.^32^

 Statin levels in blood were measured through a blood test. The most common way to measure statin levels is by measuring the level of the drug itself, which is also known as drug concentration. Enzyme-linked immunosorbent assay (ELISA) was used in this study, which utilized antibodies that are specific to the statin drug to measure the concentration of the drug in the blood. ELISA had several advantages over other methods of measuring statin levels, including its high sensitivity, specificity, and reproducibility.^33,34^

###  Statistical analysis

 Descriptive results are presented by mean (standard deviation (SD)) and frequency (percentage) for numeric and categorical variables, respectively. The normality of the variables was assessed and assured by the descriptive measures of distribution, the skewness (within ± 1.5), and the kurtosis (within ± 2). Fisher’s exact test for categorical variables and independent t-test for normal numeric variables were used to compare the clinically significant and subclinical groups.

 LPA was used to explore the latent risk profiles age, SBP, DBP, lipid profile (HDL- Cholesterol, LDL- Cholesterol, Cholesterol, and Triglyceride), FBS, insulin, BMI, and waist circumference were included in the LPA, after transforming into z-scores. To ensure the comparability of variables within the LPA model, we employed z-scores. This facilitates meaningful comparisons and analysis within the LPA framework. We fitted models with various classes, and the Akaike information criterion (AIC), Bayesian information criterion (BIC) and Adjusted Bayesian information criterion (ABIC) were utilized to compare and select the best-fitted model. The smaller values of the criteria show a better fit of the model. In addition, entropy was considered as an alternative measure to select the model. This measure ranges from 0.00 to 1.00, and higher values indicate a better model fit.

 We used MPlus 7.4^35^ for LPA and SPSS 17^36^ for all other analyses, The profile plot was drawn using Microsoft Excel 2019.

## Results

 A total of 500 participants were entered in the final analysis, with a mean age of 43.0 (SD 7.2), and a mean BMI of them was 28.1 (SD 4.0). Around 63.8% of participants were male, 89.1% as reported, non-smoker, and 9.9% as reported, past or current smoker. More detailed information about the participants’ profiles is provided in Table 1.

**Table 1:** Participants’ demographic profile

**Variables**		**n/Mean**	**%/SD**
Age (years)		43.2	7.2
Sex (male)		319	63.8
Diabetes			
	Yes	21	4.2
	No	479	95.8
Smoking			
	Past or current	49	9.9
	Never	445	89.1
Hypertension			
	Yes	38	7.6
	No	462	92.4
Statin			
	Yes	13	2.6
	No	487	97.4
Metabolic syndrome			
	Yes	114	22.8
	No	386	77.2
Waist circumference (cm)		97.7	9.4
BMI (kg/m^2^)		28.1	4.0
FBS (mmHg)		87.2	22.3
SBP (mmHg)		115.5	14.5
DBP (mmHg)		75.5	9.5
Triglycerides (mg/dL)		126.4	60.0
HDL cholesterol (mg/dL)		45.2	10.7
LDL cholesterol (mg/dL)		107.8	31.6
Total cholesterol (mg/dL)		178.3	38.7
Hemoglobin (g/dL)		15.5	1.6
Hematocrit (L/L)		44.9	4.4
Insulin (μmol/L)		9.4	8.2

BMI: Body mass index; FBS: Fasting blood glucose; SBP: Systolic blood pressure; DBP: Diastolic blood pressure; HDL: High-density lipoprotein; LDL: Low-density lipoprotein.

 The model fit indices are summarized in Table 2. LPA models with 1– to 7-class were compared for model fit. The information criteria reduced as the number of classes increased, however, the complexity of the model increased, so that the number of model parameters went up. This caused a decline in the entropy measures after 3rd class. Considering the fit indices along with the interpretability of the derived classes, we decided on LPA with tree classes. For this number of classes, there were no considerable differences between criteria and entropy than profiles with a higher number of classes. More importantly, the 3-class profile was interpretable, as well as, had reasonable prevalence of occurring. But models of 4 classes and more, had imbalance frequencies within classes (Table 2).

**Table 2:** Latent profile model fit indices

**Number of classes**	**- Log-likelihood**	**Number of parameters**	**LR Chi2(df)**	**AIC**	**BIC**	**ABIC**	**Entropy**	**class count(n)**
1-Class	8524.2	29	57.82(87)	17106.4	17228.6	17136.6	---	500
2-Class	8135.6	48	25.929(79)	16367.3	16569.6	16417.2	0.828	353/147
3-Class	**7982.3**	**67**	**26.348(71)**	**16098.6**	**16381.0**	**16168.3**	**0.803**	**212/109/179**
4-Class	7797.2	86	25.819(63)	15766.4	16128.9	15855.9	0.847	179/8/211/102
5-Class	7671.5	105	26.388(55)	15553.0	15995.6	15662.3	0.873	160/203/86/46/5
6-Class	7580.2	124	27.896(47)	15408.3	15930.9	15537.3	0.891	117/215/59/50/54/5
7-Class	7509.5	143	27.491(39)	15304.9	15907.6	15453.7	0.872	66/5/109/53/166/55/46

LR: Likelihood ratio; df: Degrees of freedom; AIC: Akaike information criteria; BIC: Bayesian information criteria; ABIC: Adjusted Bayesian information criteria. The optimal model is shown in bold font. Bold numbers show significant class based on the LPA results.

**Figure 1:**
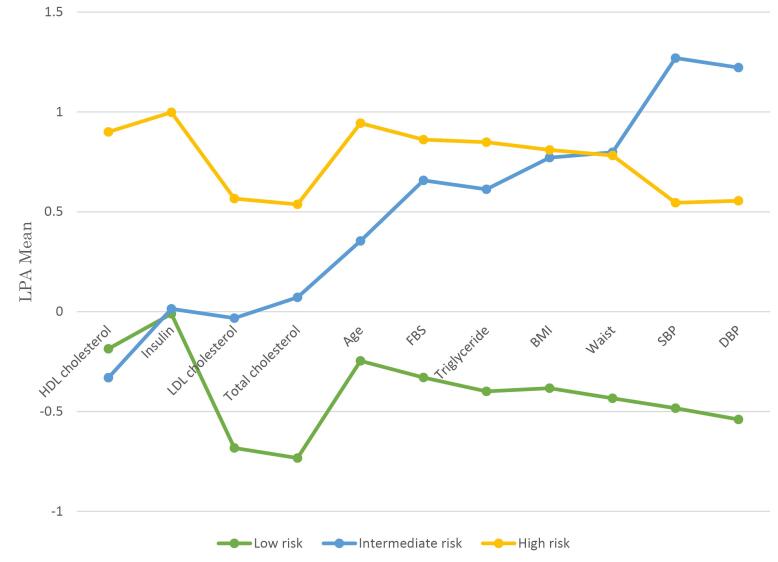
Low, intermediate, and high latent profiles of the study participants Z-scores of the variables are displayed in the profile plot

Figure 1 shows the conditional LPA means for CMetD indicators in each class. Participants in class 1, were younger individuals with lower systolic/diastolic BP, FBS, total cholesterol, LDL cholesterol, triglyceride, BMI, and waist, but had higher HDL-cholesterol. The prevalence in this class was 42.4% (212 out of 500), and we called this class a “Low-risk” latent profile. Participants in Class 2 were older compared to those in Class 1, with elevated systolic/diastolic BP, total cholesterol, LDL cholesterol, triglyceride, BMI, and waist, but had lower HDL-cholesterol. The prevalence in this class was 21.8% (109 out of 500), and we called this class an “Intermediate-risk” latent profile. Furthermore, insulin levels were similar in both classes. In class 3, variables were found to be at a higher level compared to class 2, except systolic/diastolic BP and waist. The prevalence in this class was 35.8% (179 out of 500), and we called this class a “High-risk” latent profile.

 The results of logistic regression evaluating the associations between the three LPA-driven classes and categorical variables are depicted in Table 3. Haemoglobin and hematocrit levels were significant predictors of low and intermediate latent profiles. Higher haemoglobin levels showed subordinate odds of being in low and intermediate latent profiles, while, higher hematocrit levels were associated with low and intermediate latent profiles compared to the high-risk profile (all *P*< 0.05).

**Table 3:** Results of multivariable multinomial logistic regression models for categorical factors affecting the LPA-derived classes

	**Low #**			**Intermediate #**		
**Variables**	**Adjusted odds ratio**	**95% confidence interval**	***P*** **-value**	**Adjusted odds ratio**	**95% confidence interval**	***P*** **-value**
Sex						
Female	Referent					
Male	1.361	0.714-2.598	0.349	1.506	0.651-3.478	0.339
Metabolic syndrome						
No	Referent					
Yes	0.850	0.510-1.415	0.532	0.761	0.402-1.440	0.401
Statin						
No	Referent					
Yes	2.250	0.554-9.138	0.257	1.949	0.363-10.481	0.437
Hypertension						
No	Referent					
Yes	1.050	0.456-2.417	0.910	1.775	0.715-4.407	0.216
Hemoglobin (g/dL)						
≥ 16.7	Referent					
≤ 14	0.159	0.039-0.647	**0.010**	0.043	0.008-0.247	**<0.001**
14.5-15.5	0.235	0.076-0.725	**0.012**	0.186	0.050-0.691	**0.012**
15.6-16.6	0.522	0.225-1.212	0.130	0.399	0.153-1.039	0.060
Hematocrit (L/L)						
≥ 48	Referent					
≤ 42	7.802	1.795-33.908	**0.006**	12.619	2.094-76.054	**0.006**
42.1-44.9	3.731	1.179-11.801	**0.025**	3.260	0.849-12.525	0.085
45-47.9	1.070	0.458-2.500	0.875	1.168	0.447-3.053	0.752

^#^ The high-risk class is considered a reference class. The significant results are shown in boldface font.

## Discussion

 This study used baseline data for 500 healthcare providers to extract latent profiles based on the risk factors of CMetDs. We identified three latent profiles of CMetD risk factors, low, intermediate, and high-risk profiles among healthcare providers. Given that different population groups (health care providers), they are unique as expected they should have a healthier lifestyle compared to the public population. Therefore, the low-risk group had the highest number of class members with more than 40% of the whole sample. Our results showed that the low-risk profile had a higher prevalence of HDL cholesterol, and insulin, which have been identified in the literature as definite CMetDs risk factors.^37,38^ In this study, the high-risk profile was characterized by severe risk factors such as lipid profile, FBS, insulin, age, BMI, and waist circumference in CMetDs, which were less healthy compared to the intermediate-risk profile. There are only a few studies that have used LPA to detect the latent profiles of CMetDs.^39-41^

 Our findings suggest that exposure to the high-risk profile impacted the health of healthcare providers from early youth to late adulthood. Although participants with high-risk profiles had higher conditional probabilities for all the CMetD risk factors, the largest difference between the CMetD subtypes was found for SBP and DBP in the intermediate-risk group. In the study by Verswijveren et al^42^ three latent profiles were identified to assess the effect of activity accumulation on CMetD risk factors in youth, with waist circumference, SBP, and DBP identified as high-risk factors among these individuals. In the study by Jenkins,^29^ four latent profiles were identified to examine the CMetD’s correlates of physical activity and sedentary patterns in US youth, with waist circumference, SBP, and DBP being important predictors of CMetDs. In previous studies, the association between BP and CMetDs has been well-established,^43,44^ and specific guidelines have been developed for the identification and management of CMetDs in BP care.^44,45^ Our results emphasize the importance of implementing these integrated care guidelines.

 Our results showed that individuals in the high-risk profile had older average age, higher insulin, triglycerides, total cholesterol, FBS, and BMI. Previous studies have demonstrated high-risk profiles in CMetDs, which are characterized by specific biomarkers and physiological parameters such as weight, BMI, waist circumference, BP, FBS, and dyslipidemi.^38,41,46^ Furthermore, these studies included more biological indices such as waist-to-height ratio, body fat index, overweight, and obesity to identify high-risk characteristics in CMetDs. These longitudinal studies support the hypothesis that behavioural factors, dietary improvements, prevention of weight gain in adults, and prevention of overweight in adolescents are key factors in reducing CMetDs and the risk of developing them. Our cross-sectional study is limited to data collected from healthcare providers, without considering participants’ lifestyle and socio-economic status, which may lead to gaps in our understanding of CMetDs mechanisms.

 Similarly, in the Caspian study^47^ conducted on students aged 7-18 from 30 provinces in Iran, four latent classes of CMetDs components were identified; healthy (59.6%), low risk (20.4%), moderate risk (13.7%), and high risk (6.4%). In the high-risk group, individuals have high levels of LDL cholesterol and total cholesterol, while in the low-risk group, they have high levels of HDL cholesterol. Another study conducted among Iranian adults over the age of 20 who visited the endocrine centre at Tehran University of Medical Sciences demonstrated the existence of four latent classes of CMetDs components; non-Mets (38.4%), low risk (18.6%), high risk (24.2%) and Mets (18.7%). Among these four classes, the high-risk class was associated with high waist circumference, SBP, and DBP.^48^ However, latent profiles among Iranian children and adults and our study have revealed a different pattern of CMetD pattern among children and those we have found among adults. This difference could be attributed to sample size, study location and conditions, participant age, and other unknown factors. Additionally, differences among different generations may be an explanation for the varieties.

 While the three-class model was selected based on statistical indicators such as AIC, BIC, ABIC, and entropy, we acknowledge that models with more classes exhibited imbalanced class sizes, which can impact the robustness and power of analysis. For instance, in the four- to seven-class solutions, one or more classes comprised fewer than 10% of the total sample, raising concerns about model stability and generalizability. Thus, although these models demonstrated slightly improved statistical fit, the practical interpretability and prevalence of small subgroups limited their utility. In contrast, the three-class solution provided a more balanced and interpretable classification of risk profiles, with each class containing a sufficiently large portion of participants to allow for meaningful comparisons. Therefore, the decision to adopt the three-class model reflects a deliberate balance between statistical rigor and clinical relevance, ensuring both interpretability and analytic strength.^24^

 The present study showed significant associations between risk factors and the LPA-driven profiles. Furthermore, according to the results of multivariable logistic regression, being male, the presence of metabolic syndrome, statin use, and the presence of hypertension were not associated with the related risk profiles. The lack of a significant association for these variables may reflect the relatively homogeneous and health-conscious nature of the study population, as well as potential limitations in statistical power due to the moderate sample size. However, lower levels of hematocrit and hemoglobin were significantly linked with low and moderate risk profiles. In the study by Anavekar,^49^ lower hemoglobin concentration and being male were identified as risk factors for CMetDs in the univariate analysis. In study by Kunnas et al^50^ suggested that hematocrit be included in the CHD risk factor profile in Finnish men over 55 years of age. Furthermore, a study conducted on individuals aged 30 to 75 from the United States of America identified a relationship between hematocrit levels and CMetD risk factors.^51^ Our findings, along with those of others, have shown that in various populations, including both youth and adults, different levels of hemoglobin and hematocrit have been identified as influential risk factors for CMetDs.

 While the observation that lower hemoglobin and hematocrit levels are significantly associated with low- and moderate-risk profilesmay seem counterintuitive in light of previous research that identified low hemoglobin or hematocrit as potential risk factors for cardiometabolic diseases (e.g., Anavekar et al 2004;⁴⁹ Kunnas et al 2009;⁵⁰ Brown et al 2001⁵¹), it is important to consider the unique characteristics of our study population—healthcare providers, who may be relatively healthier and more health-conscious compared to the general population. Additionally, the cross-sectional design limits our ability to infer causality. It is also possible that individuals in the high-risk group had elevated levels of hemoglobin and hematocrit due to compensatory physiological mechanisms related to obesity, insulin resistance, or metabolic syndrome, as reported in previous studies (e.g., Pi-Sunyer, 2002⁵^2^). These findings highlight the complex and context-dependent relationship between hematologic indices and cardiometabolic risk, suggesting the need for further longitudinal and mechanistic research to clarify these associations.

 Our study demonstrated that for Iranian adults, BMI and waist circumference are likely to be the primary predictors associated with the risk of CMetDs. Unmeasured lifestyle and environmental factors in our study may have affected higher BMI and waist circumference in adults or other risk factors for CMetDs between healthcare providers. The influence of environmental, behavioural, psychosocial, and genetic factors on high BMI is well recognised.^52,53,54^ In the study conducted by Borges et al^28^ predictive analysis of early development of CMetD risk factors, revealed a significant number of overweight or obese adolescents who are associated with CMetD risk profiles. Additionally, in the study conducted by Sodjinou et al^55^ older women were significantly more at risk for CMetDs with higher BMI and waist circumference.

 The findings of this study should be interpreted considering several limitations. Firstly, due to the cross-sectional nature of this study, it is not possible to assess the temporal relationships and the transition effect of the risk factors on the latent risk profile. Utilizing the techniques of this study could provide a platform to find the transition effect of the risk factor in a cohort study, and longitudinal studies are necessary to confirm these findings. Secondly, the study population was healthcare employees in TBZMED, in northwest Iran. Therefore, the findings may not apply to the Iranian population. Studies among different populations across the country may reveal different patterns of latent profiles. Also, the study may probably lack statistical power due to the moderate sample size, so, modelling across a larger sample size may be the solution to find more reliable profiles. In addition, As the CMetD risk factors can be influenced by sex and age, larger studies can provide us with a context to assess the risk profile amongst the sub-groups, for instance, age/sex-specific latent profiles. In line with the previous limitation, larger studies are strongly required to carry out subgroup LPA within categories of some important groups. Despite these limitations, our study provided a useful finding on the pattern of shared CMetD risk factors among healthcare providers of TBZMED.

## Conclusion

 In conclusion, our study demonstrated the existence of three shared characteristics of the CMetDs risk profile among healthcare employees: low, intermediate, and high-risk profiles. Specifically, we found that higher BMI levels, higher triglyceride values, higher insulin, higher FBS, and lower SBP/DBP values were the main predictors of CMetD’s high-risk profile. The intermediate risk profile has a higher risk of waist circumference, SBP, and DBP of developing a CMetD risk profile, especially in older male employees. Conducted as part of the AZAR cohort, this study highlights the importance of designing longitudinal studies to estimate the incidence of CMetDs and monitor changes in latent profiles over time. The LPA-derived risk profiles and their predictors can enhance prevention strategies and deepen our understanding of CMetD risk factors. Older males with higher lipid profiles, BMI, insulin, and FBS levels should be screened more carefully. These findings may inform public health policies aimed at early prevention of CMetD risk factors.

## Competing Interests

 The authors declare that there is no conflict of interest.

## Ethical Approval

 The institutional review board of Tabriz University of Medical Sciences approved the protocol of the study (ethics approval number: IR.TBZMED.REC.1400.1006).
